# Conflict experience and resolution underlying obedience to authority

**DOI:** 10.1038/s41598-023-38067-z

**Published:** 2023-07-10

**Authors:** Felix J. Götz, Vanessa Mitschke, Andreas B. Eder

**Affiliations:** 1 Institute of Psychology, University of Regensburg, Universitätsstraße 31, 93053 Regensburg, Germany; 2 Georg Elias Müller Institute of Psychology, University of Göttingen, Göttingen, Germany; 3 Department of Psychology, University of Würzburg, Würzburg, Germany

**Keywords:** Psychology, Human behaviour

## Abstract

Definitions of obedience require the experience of conflict in response to an authority’s demands. Nevertheless, we know little about this conflict and its resolution. Two experiments tested the suitability of the ‘object-destruction paradigm’ for the study of conflict in obedience. An experimenter instructed participants to shred bugs (among other objects) in a manipulated coffee grinder. In contrast to the demand condition, participants in the control condition were reminded of their free choice. Both received several prods if they defied the experimenter. Results show that participants were more willing to kill bugs in the demand condition. Self-reported negative affect was increased after instructions to destroy bugs relative to other objects (Experiments 1 and 2). In Experiment 2, compliant participants additionally showed an increase in tonic skin conductance and, crucially, self-reported more agency and responsibility after alleged bug-destruction. These findings elucidate the conflict experience and resolution underlying obedience. Implications for prominent explanations (agentic shift, engaged followership) are discussed.

## Introduction

To this day, obedience is among the most broadly discussed^1,2^ but at the same time ethically most challenging topics in psychology. Obedience is defined as a person’s willingness to accommodate the demands of a legitimate figure of authority^3^ even to the extent that these conflict with their own values and norms^4,5^. Thus, obedience is a special case of compliance, which in turn comprises all kinds of favorable responses to another individual’s request^6^. The typical psychological ingredients of obedience are therefore (a) a legitimate authority figure, (b) a demand that is perceived as immoral, (c) the experience of conflict in response to the demand, and (d) the resolution of that conflict in compliance with the authority^7^.

In psychological research, the phenomenon of obedience is inextricably linked with the studies of Stanley Milgram^8–10^. Milgram (1974) reported that most people administered a potentially lethal electric shock to another person when instructed to do so by an experimenter, i.e., they violated the norm not to harm (and/or endanger) other human beings on behest of a ‘scientific authority’. In a teacher-learner arrangement, participants acted as a teacher who punished incorrect answers of a confederate (the learner) in a word-recall task via administration of increasingly severe electric shocks (ranging from 15 V to potentially lethal 450 V). However, no shocks were administered to the confederate (the learner), who reacted to the supposed jolts at predefined intensities with pre-recorded verbal responses of increasing vigor. If the teacher hesitated to administer a shock to the learner, the experimenter pressured them to proceed with pre-specified prods. While some participants refused to administer shocks, most participants (65% on average) progressed to the maximum voltage level. The original finding was reproduced by other laboratories using similar paradigms^11,12^ or variants (e.g., with a lower maximum shock level of non-lethal 150 V^13,14^ or in virtual-reality^15^). While numerous studies have investigated moderating factors of obedience (e.g., legitimacy^16^, conscientiousness^17^) and suggested psychological processes^18–20^ involved in obedience, the underlying conflict experience and its resolution has received much less attention in psychological research^21,22^.

Experiencing conflict between one’s personal values and norms and an authority’s immoral demands is a crucial requirement of obedience because it is the need to resolve this conflict that makes obedience a special case of compliance^4^^,cf.^^6^^.^ Possible psychological processes underlying ‘obedient conflict resolution’ are described by two accounts that make predictions regarding associated affective response characteristics: Milgram's original agentic shift account^4^ and Haslam and Reicher's engaged followership account^22^. While both accounts agree in assumptions about an initial conflict experience of the individual, they differ in their explanation of the psychological processes that resolve the conflict: Milgram proposed a shift from a self-directed mode to an ‘agentic state’, in which the individual dissociates from the consequence of the demanded actions and rejects responsibility for their outcome. Here, the initial negative affect is proposed to shift to a neutral affective state during obedience but to return after task completion. By contrast, Haslam and Reicher suggested a process of social persuasion, in which the social identification with the experimenter’s scientific enterprise generates compliance with his destructive demands. Here, the initial negative affect is proposed to shift to an increasingly positive state, especially after task completion. Empirically, however, both accounts had to deal with both supportive and difficult-to-reconcile findings and arguments. Regarding the agentic-shift account, one study found that acting on commands rather than on one’s own initiative decreased the sense of agency^23^. However, a re-analysis of Milgram’s archived data revealed that the mean shock level of skeptical participants was higher than that of participants who believed in the deception^24^. Regarding the engaged-followership account, one study showed that taking part in a prototypical rather than a non-prototypical science experiment mostly increased obedience^25^. However, another re-analysis of Milgram’s archived data indicated that only a small number of obedient participants explained themselves by identifying with science^18,26^.

To elucidate the conflict experience and resolution associated with obedience, two methodological challenges must be overcome. First, participants must be confronted with a demand that is perceived as immoral by many. However, pressuring participants into (allegedly) administering dangerous electric shocks to other human beings was^27^ and is problematic because it violates ethical standards in psychological science^28^. Second, to test participants' conflict experience and show that its resolution is a case of obedience, two types of control conditions are needed: (1) Participants willingness to comply with the immoral demand must be compared with a control group in which they are *asked* rather than demanded to do so. (2) Individuals’ affective response characteristics in response to the immoral demand, especially following conflict resolution, need to be systematically registered and compared to those in response to unproblematic control demands.

## The present research

To confront participants with an immoral but ethically more acceptable conflict, the present research tested a novel adaptation of the bug-killing paradigm^29^: the ‘object-destruction paradigm’. In the bug-killing paradigm, participants are instructed by an experimenter to kill bugs by supposedly shredding them in a (manipulated) electric coffee grinder. In fact, however, no bug comes to harm. Previous research has already used the paradigm to investigate the effects of initial bug-killing^30^, synchronization^31^, or gratitude (in a worm-killing variant)^32^ on *subsequent* volitional or voluntary killing, in the latter two instances as an index of obedience. Indeed, killing bugs is in conflict with norms to respect animal life, because for most adults^33^ bugs are living beings and harming or killing insects is not acceptable^34^. Furthermore, killing bugs is an irreversible act whereas the administration of non-lethal electric shocks in Milgram’s paradigm^4^ and modern variants^13^ is a reversible noxious act. Thus, it is plausible that many participants would experience conflict with an experimenter’s bug-killing demand. Nevertheless, intentionally killing a bug is likely to be less distressing and traumatic for the participant than torturing (and/or potentially killing) a fellow human being. In fact, a recent study showed that people consider it more permissible to harm animals, and especially inanimate objects, due to speciesism (i.e., their non-human species membership) and the belief that they suffer less and have lower cognitive capacity^35^. Therefore, we consider the bug-killing paradigm to be less problematic in terms of violating ethical norms.

To shed light on obedient individuals’ conflict experience and resolution, the object-destruction paradigm comprises a demand and a control condition, in which the experimenter explicitly reminded participants of their free decision. The latter condition was introduced to assess participants’ readiness to kill the bugs without a clear demand from the experimenter, thus testing participants’ conflict experience directly. Moreover, the paradigm comprised several control destruction tasks to investigate affective response characteristics specific to allegedly killing bugs. More precisely, participants were instructed to destroy inanimate objects (e.g., coffee beans) in addition to bugs, supposedly for the scientific investigation of ‘feelings of destruction’ (cover story). Thus, the destruction tasks allow for assessments of the aversiveness of the bug-killing task. If participants refused the experimenter’s demands, he had two pre-determined prods to pressure them to continue. For an explicit measurement of their affective state after conflict experience, participants reported their feelings of pleasure, arousal, and dominance following each destruction task. Experiment 2 elucidated participants’ conflict resolution in more detail: They additionally reported feelings of agency and responsibility and tonic skin conductance levels (SCL) were measured. We expected more participants to destroy the bugs in the demand than the control condition. Moreover, participants should experience more tension following the bug-destruction task as compared to the other destruction tasks, indexed by increases in self-reported negative arousal.

## Experiment 1

Experiment 1 assessed whether the ‘object-destruction paradigm’ is effective in generating obedience to bug-destruction instructions against participants’ desires to spare the bugs’ lives. We hypothesized that participants in the demand condition would comply more with the experimenter’s bug-killing demands compared to participants in the control condition. Furthermore, participants in both conditions should experience inner conflict triggered by the experimenter’s immoral instruction to kill a live animal, as indexed by higher ratings of negative affective arousal after the instructed destruction of live bugs. For a closer look on compliant participants, we additionally conducted affective response analyses excluding defiant participants.

## Methods

### Participants

Forty-five participants (34 females, *M* = 31 years, *SD* = 12.17, range 18–65 years) volunteered for a financial compensation of 5 Euros. A sensitivity analysis (calculated with G*Power 3.1^36^) revealed that a sample size of 45 participants had sufficient statistical power of 1 − β = 0.80 to detect effects with w ≥ 0.42 in a Chi-square goodness of fit test with condition (demand, control) as variable. Thus, we aimed for a strong effect to make the paradigm interesting for follow-up research. The study protocols were approved by the local research ethics committee of the Institute of Psychology of the University of Würzburg (ethics approval number: GZEK 2016-09). Both experiments were carried out in accordance with the approved protocols, the Helsinki declaration on ethical research with human participants, and the guidelines for ethical research with humans from the German Psychological Society (DGPs). Reporting follows as far as possible and necessary the recommendations of the ARRIVE guidelines^37^.

Participants were recruited via the participant pool management software (SONA) of the Department of Psychology at the University of Würzburg. The study description contained three explicit warnings: First, potential participants were warned that the study would include tasks that could trigger negative feelings. Second, potential participants were informed that each experimental session would be recorded with a video camera. Third, potential participants were warned that they would have to move test tubes containing live bugs, albeit without direct bodily contact with the bugs. Psychology students were not allowed to participate.

### Materials

**Figure 1:**
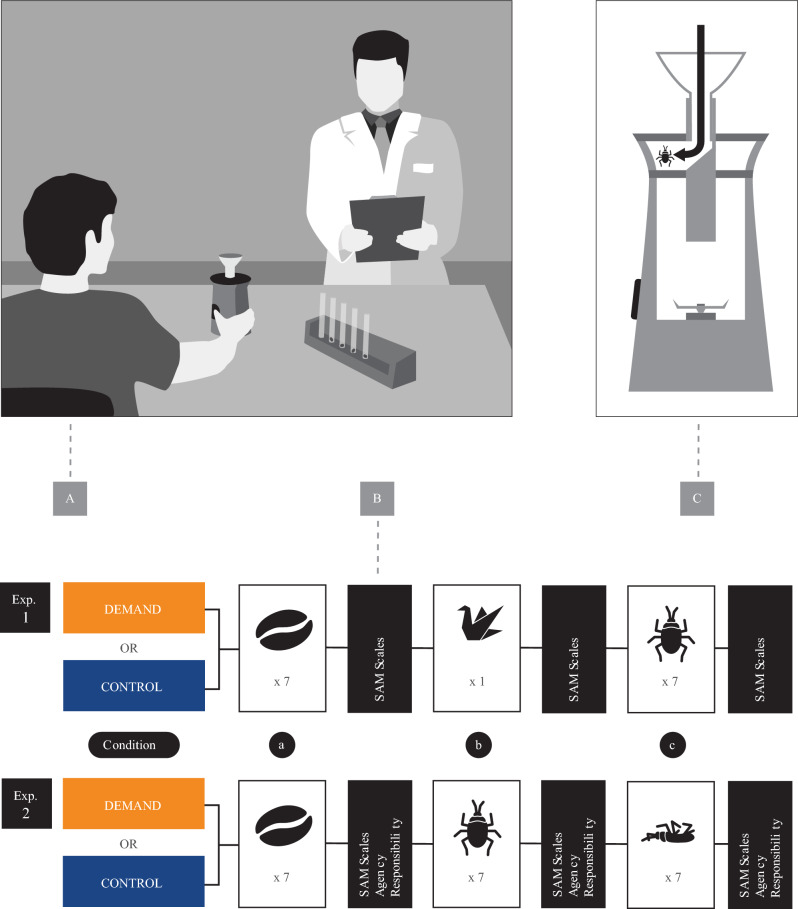
Panel (**A**) shows the experimental situation with the participant sitting in front and the experimenter standing behind the desk. Panel (**B**) visualizes the three destruction tasks of Experiments 1 and 2, including the following self-report measures. Panel (**C**) shows a cross-section of the ‘Destruction Machine’ to visualize the double-bottom manipulation. Adobe Illustrator (version 27.1; https://www.adobe.com/products/illustrator.html) was used to create all images, arrange all panels, and edit plots and texts.

The ‘destruction machine’ was a modified electric coffee grinder with a mounted tube on top and a funnel within the tube as well as an invisible double bottom hidden within the lid (for a visualization, see Fig. 1C). The destruction machine was used for all three destruction tasks.

The bugs were raised and fed in the laboratory rooms. Specifically, one package of mealworms was purchased from a pet store, raised to flour beetles in a terrarium in a species-appropriate manner, and cared for regularly (feeding, regular cleaning of the terrarium, etc.). The dead bugs used in Experiment 2 had died naturally and were temporarily stored in a freezer for later experimental use. After completion of Experiment 2, all remaining bugs were released into the wild.

### Procedure

When participants arrived in the laboratory (one at a time), they were told that the study investigates “what destruction feels like” (cover story). Then, the ‘destruction machine’ was presented and its functioning was explained in detail. In addition, the experimenter repeated the warnings from the study description and reminded them of their right to exit the study at any point without giving reasons. After providing informed consents to participation in the study and video recordings, they were asked to follow the instructions on a laptop. The experimenter then started the video recording. Instructions on the laptop first familiarized the participant with the 9-point Self-Assessment Manikin (SAM) scales that were presented after each destruction task to indicate feelings of dominance, pleasure, and arousal^38^. Before the first destruction task, participants indicated the extent of their moral circle with respect to a list of 28 entities (among them animals like *bugs* as well as plants like *coffee beans*). More precisely, they decided for each single entity if they regarded them worthy of moral consideration or not using single-choice yes–no-checkboxes^39^. Then, participants signaled the experimenter to continue with the first destruction task.

For the first destruction task (grinding of coffee beans), the participant was to pour coffee beans into the tube via the funnel, from which they fell into the double bottom of the device (of which the participant was unaware; see Fig. 1A-C). The participant then ground the coffee beans that the experimenter had placed in the grinder before the participant arrived in the laboratory. After grinding the beans, the experimenter opened the grinder in front of the participants with the pulverized coffee beans visible inside. The experimenter emptied the grinder into a waste bin next to the table. For the second destruction task, the participant was to place a hand-made paper crane directly into the hopper. For the third and final destruction task (bug killing), the experimenter handed over 7 flour beetles in test tubes with the instruction to destroy them one after the other. Participants were asked to pour a single bug into the tube of the destruction machine (again leading to the secret double bottom, preserving the beetles from harm without the participants’ knowledge) and to press the button of the machine for 2 s before continuing with the next. Instructions were to continue with this procedure until each of the seven bugs was killed. The time frame between the presentation of the test tubes containing the bugs and the destruction of the first bug (if compliant) and/or skipping to the SAM scales (i.e., participants’ reflection time during the bug-destruction task) was *M* = 42 s, *SD* = 23 (range 15–113 s). The time frame between the presentation of the beans and participants’ responses to the SAM scales following the bug-destruction task (i.e., the time frame of all three destruction tasks combined) was *M* = 267 s, *SD* = 43 (range 191–393 s).

After each destruction task, participants reported current feelings of pleasure, arousal, and dominance on SAM scales^38^. The last SAM rating was followed by several questionnaires. The questionnaires were (sorted according to the order of appearance): Situational Self-Awareness Scale^40,41^, competence and warmth judgments with respect to the experimenter^42^, Belief in Science Scale^43,44^, 10-item version of the Big Five Inventory^45^, Short Sadistic Impulse Scale^46^, the Revised Cheek and Buss Shyness Scale^47^. Data and analyses of all questionnaires are accessible in the OSF repository. Ratings and questionnaires were provided on a laptop in the laboratory room. The experimental code was programmed using Inquisit 3 software. At the end, participants were thoroughly debriefed about the manipulated coffee grinder and the deception. The experimenter made sure that each participant understood that no animal had been harmed and that they did not need to worry about their behavior during the bug-destruction task.

### Demand vs. control condition

Participants were randomly assigned to one of two conditions. In the demand condition, the experimenter merely instructed participants to destroy the beans, crane, and live bugs. If a participant expressed concerns or refused to follow the instruction, two prods were given. Both prods were formulated in such a way that participants’ freedom of choice was not explicitly compromised at any time^cf.^^4^. After the first complaint/refusal, the prod was: “Follow my instructions. I am responsible”. After the second complaint/refusal, the prod was: “The purpose of the experiment is to find out what destruction feels like. Therefore, it is necessary that you proceed”. If participants refused a destruction after these prods, the experimenter told them to skip the respective destruction task and to continue with the SAM scales. In the control condition, instructions and prods were almost the same except that the experimenter additionally stressed the participants’ free decision at the end. Specifically, the first prod was: “I want to emphasize: I am responsible. The decision is yours”. The second prod was: “The purpose of the experiment is to find out what destruction feels like. Therefore, it would be necessary for you to continue. However, you decide if you want to continue or not”. All instructions and prods were given verbally in a matter-of-fact tone by a trained male experimenter in a highly standardized way. We decided to use two prods (and not four prods as in the Milgram Studies^4^) to ensure that participants understood that the experimenter really wanted them to continue (and not covertly the opposite^cf.^^48^). The exact instructions (and/or their English translations), the research protocols, and detailed transcripts of the video-recorded bug-destruction tasks are accessible in the OSF repository.

## Results

### Number of compliant vs. defiant participants

**Figure 2:**
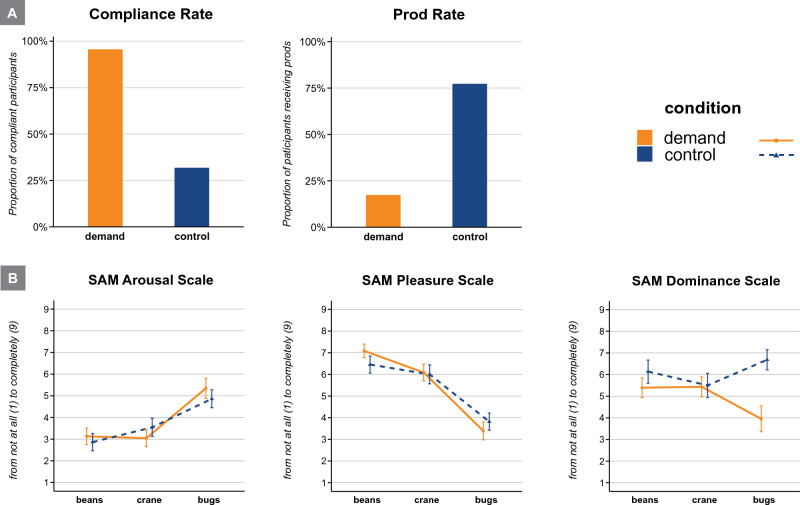
Results of Experiment 1. Panel (**A**) shows the proportion of compliant participants and the proportion of participants that had received prods per condition in per cent. Panel (**B**) shows self-reported levels of arousal, pleasure, and dominance per destruction task and condition. All error bars indicate standard errors.

The proportion of participants who complied versus defied the experimenters’ bug-destruction instruction in each condition were submitted to a chi-squared test of association for independent samples. The difference between the conditions was significant, χ^2^(1, *N* = 45) = 20.00, *p* < .001, Φ = 0.667 (see Fig. 2A). In the demand condition, 22 out of 23 participants (96%) killed the bugs, whereas in the control condition only 7 out of 22 participants (32%) followed the experimenter’s instruction. By contrast, the coffee beans were destroyed by all participants in both conditions and the paper crane was spared by only one single participant in the control condition.

### Number of prods

Proportions of participants in each condition that received a prod were submitted to a χ^2^-test of association for independent samples. The difference between conditions was significant, χ^2^(1, *N* = 45) = 16.20, *p* < .001, Φ = 0.600 (see Fig. 2A). In the demand condition, 19 out of 23 participants (82.6%) were not prodded; one participant received one prod (4%) and three participants received both prods (17.3%). In the control condition, 5 out of 22 participants (22.7%) received no prod and seventeen (77.3%) received both prods.

### Self-reported affect

Means of self-reported feelings of pleasure, arousal, and dominance were each submitted to 2 × 3 mixed analyses of variance (ANOVA) with *Condition* (demand vs. control; between) and *Destruction Task* (coffee beans vs. paper crane vs. live bugs; within) as factors. In the ANOVAs regarding feelings of pleasure and arousal, the main effects of *Destruction Task* were significant: Participants reported more arousal, *F*(2,86) = 32.85, *p* < .001, η_*p*_^2^ = 0.43, and less pleasure, *F*(2,86) = 54.31, *p* < .001, η_*p*_^2^ = 0.56, after the bug-destruction task relative to the other two destruction tasks (coffee beans, paper crane; see Fig. 2B). The ANOVA of dominance ratings yielded a main effect of *Condition* and an interaction effect of *Condition* and *Destruction Task*. Overall, participants in the demand condition felt less dominant relative to the control condition, *F*(1,43) = 4.36, *p* = .043, η_*p*_^2^ = 0.09. This difference was especially pronounced after the bug-destruction task, *F*(2,86) = 6.01, *p* = .004, η_*p*_^2^ = 0.12 (see Fig. 2B).

The effects regarding arousal and pleasure were also significant in sub-analyses with compliant participants only (i.e., 22 participants in the demand and 7 in the control condition): Compliant participants felt more aroused, *F*(2,54) = 20.88, *p* < .001, η_*p*_^2^ = 0.44, and less pleased, *F*(2,54) = 39.17, *p* < .001, η_*p*_^2^ = 0.59, subsequent to bug-destruction.

All other tests of the ANOVAs were above the significance level, *F*s ≤ 2.63 and *p*s ≥ .116.

### Questionnaires

The means of the questionnaires were also submitted to t-tests with condition as between-participants factor. Results were above the statistical significance level and are accessible in the OSF repository.

## Discussion

In line with Milgram’s definition of obedience^4^, the results of Experiment 1 indicate that many participants experienced conflict when instructed to destroy live bugs. Most participants in the demand condition chose to resolve the conflict in accordance with the experimenter’s demands (i.e., they were obedient) whereas most participants in the control condition chose to defy them. Moreover, participants self-reported more negative arousal after the bug-destruction task as compared to the other two destruction tasks, irrespective of condition. Interestingly, participants in the demand condition felt less dominant subsequent to alleged bug-killing than participants in the control condition. These results are both in line with a process of an initial conflict experience that reemerges after task realization (i.e., the predictions of the agentic-shift account^4^) and a process of social persuasion accompanied by an affective shift from an initially negative to an increasingly positive state (i.e., the predictions of the engaged followership account^22^).

## Experiment 2

Experiment 2 modified the object-destruction procedures of Experiment 1 to investigate obedient participants’ conflict resolution in more detail. In line with the conflict experience hypothesis, we expected more participants to destroy the live bugs in the demand vs. control condition. Moreover, we expected participants to experience more stress subsequent to the instruction to kill live bugs relative to other objects, indexed via a decrease in pleasure, and increases in arousal. To investigate how participants reflected their part in the alleged bug-killing, participants self-reported estimates of agency and responsibility after each destruction task ^e.g.,^^49^. Here, the agentic-shift account^4^ predicts that obedient participants should feel less responsible for the destruction of the bugs in the demand as compared to the control condition based on a ‘transfer of responsibility’ from the participant to the experimenter. Note that the engaged followership account does not make explicit assumptions with respect to responsibility and/or agency^22^. To exploratorily investigate compliant vs. defiant participants’ negative arousal during the bug-killing demands and following their realization or refusal in a physiological manner, tonic skin conductance levels (SCLs) were measured during instruction and after the bug-destruction task. Here, the agentic-shift account predicts that obedient participants’ initial conflict experience returns (i.e., larger SCLs)^4^. To test the effectiveness of the bug-killing deception, participants were asked about their suspicions in an extended debriefing about the experimental procedure ^cf.^^24^. To control for sequence effects (e.g., due to acting consistently) and to examine whether the disobedience observed in Experiment 1 was specific to the demand of killing *live* bugs, participants were instructed to destroy dead bugs after they had been instructed to destroy live bugs.

## Methods

### Participants

Sixty-two participants (39 female, 23 male, *M* = 25.05 years, *SD* = 4.94, range 18–50 years) volunteered for a financial compensation of 7 Euros. Participants were recruited via SONA using the same restrictions as for Experiment 1. Participants from Experiment 1 were not recruited. Sample size planning was based on a sensitivity analysis with a sample of *N* = 60. With respect to the Chi-square goodness of fit tests regarding the numbers of compliant participants and prods per condition, this sample size was sufficient to detect a medium-sized effect of w ≥ 0.36 with a probability of 1 − β = 0.8 (calculated with G*Power 3.1^36^). We recruited 62 participants from our laboratory subject pool to compensate for possible dropouts. One participant was excluded from the analyses because the experimenter identified her as a psychology student. The study procedure was approved by the local ethics committee (GZEK 2016-09).

### Material and procedure

Materials and procedure of Experiment 2 were identical to Experiment 1, with the following exceptions: (1) Participants were instructed to destroy the coffee beans one at a time instead of all at once. (2) The destruction of a paper crane was removed from the procedure. Note that to increase the noise produced by the coffee grinder, the experimenter secretly planted bits of paper within the hopper while emptying the grinded coffee beans into the bin^29^. (3) Participants had to additionally destroy dead bugs after they had destroyed (or had refused to destroy) live bugs. In consequence, beans and bugs were destroyed in the following order: coffee beans, live bugs, dead bugs. (4) Participants reported their feelings of agency and responsibility after each destruction task and subsequent to the SAM ratings^38^. Specifically, participants indicated their proportion of active contribution to the destruction of all entities (coffee beans, live bugs, dead bugs) in percent as a measure of agency^cf.^^23,50^. To assess self-perceptions of responsibility, participants were to use a 9-point self-assessment scale (1 = *no responsibility*, 9 = *full responsibility*). (5) Tonic skin conductance levels (SCL) were measured while video recording of the destruction tasks was dropped. (6) The experimenter asked all participants if they believed that they had destroyed the bugs during an extended debriefing. The experimenter began by asking a broad question, inquiring whether there were any suspicions regarding the experiment. Following this initial query, the experimenter proceeded to ask a series of increasingly specific questions regarding both the general procedure and the task of bug destruction in particular (cf. funnel debriefing^51^).

### SCL measurement

At the beginning of the session and before the first destruction task, two electrodes were attached to the index and middle finger of the participant’s non-dominant hand. The use of the electrodes was justified by the cover story (i.e. the supposed scientific investigation of ‘feelings of destruction’). For the establishment of a baseline measurement, a two-minute resting period was included at the start of the experiment during which the participant was instructed to sit calm and relax. Three critical points in time were identified in advance and marked by the experimenter by manual triggers for the analyses: (1) Start of the destruction instruction, (2) start of the alleged destruction, and (3) end of the destruction task. Due to the short duration of the original baseline period and a constant drift in the tonic skin conductance (likely due to a high room temperature; Experiment 2 was conducted in summer), we decided to focus on the comparison between small time frames that are similar in length between compliant and defiant participants, namely start of the bug-destruction instruction, end of the bug-destruction task, start of the dead bug-destruction instruction. Tonic SCL was calculated via a continuous decomposition procedure^52^, using LedaLab Version 3.4.9. To assure that data points are comparable between participants (although overall timing may vary), SCL levels were averaged across a time bin of 30 s directly following each significant event (Instruction, Destruction Task End, next Instruction).

## Results

### Number of compliant vs. defiant participants

**Figure 3:**
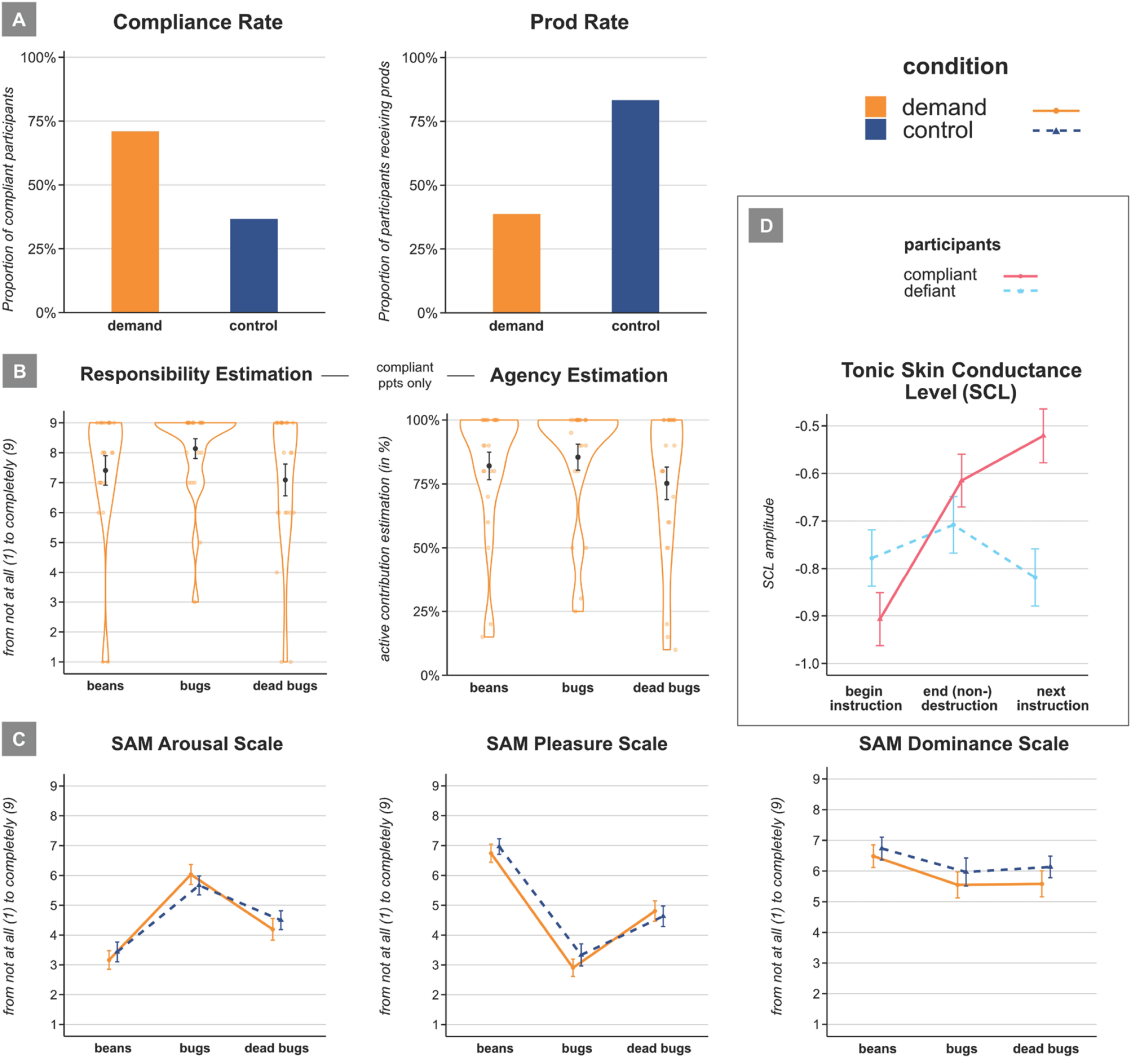
Results of Experiment 2. Panel (**A**) shows the proportion of compliant participants and the proportion of participants that had received prods per condition in per cent. Panel (**B**) shows only compliant participants’ (*n* = 33) self-reported responsibility and agency estimates. The small dots indicate the individual responses of the participants. Panel (**C**) shows self-reported levels of arousal, pleasure, and dominance per destruction task and condition. Panel (**D**) shows tonic skin conductance levels (SCL) of compliant vs. defiant participants during and after the bug-destruction task. SCL amplitude was averaged over 30 s. Error bars indicate standard errors.

The numbers of participants that complied with vs. defied the experimenters’ bug-destruction instruction (live bugs, dead bugs) per condition were submitted to χ^2^ tests of association for independent samples. The difference of the first test was statistically significant, χ^2^(1, *N* = 61) = 7.22, *p* = .007, Φ = 0.344 (see Fig. 3A). Twenty-two out of 31 participants (71.0%) allegedly killed the live bugs in the demand condition, whereas only 11 out of 30 participants (36.7%) did so in the control condition. One participant in the control condition refused to continue after having destroyed a single bug and another participant in the control condition after having destroyed three live bugs. Both were counted among the defiant participants. The dead bugs were destroyed by 25 out of 30 participants (83.3%) in the control condition and 30 out of 31 participants (96.8%) in the demand condition, χ^2^(1, *N* = 61) = 3.11, *p* = .078, Φ = 0.226. The coffee beans were destroyed by all participants.

### Number of prods

The numbers of participants in each condition that received a prod during the bug-destruction tasks (live bugs, dead bugs) were submitted to χ^2^ tests of association for independent samples. The difference of the first test was significant, χ^2^(1, *N* = 61) = 16.9, *p* < .001, Φ = 0.527 (see Fig. 3A). In the demand condition, 19 out of 31 participants (61.3%) received no prod, two received one prod (12%), and 12 participants received both prods (38.7%). In the control condition, 5 out of 30 participants (16.7%) received no prod and 25 received two prods (83.3%). With respect to the destruction of dead bugs, 1 out of 31 participants (3.2%) received one prod in the demand condition and 6 out of 30 (20%) in the control condition, χ^2^(1, *N* = 61) = 4.22, *p* = .040, Φ = 0.263. No participant refused to destroy the coffee beans.

### Self-reported affect

Means of self-reported feelings of pleasure, arousal and dominance were submitted to 2 × 3 mixed-model ANOVAs with *Condition* (demand, control) as between-participants factor and *Destruction Task* (coffee beans, live bugs, dead bugs) as within-participant factor. All ANOVAs yielded main effects of *Destruction Task*: Relative to the other two destruction tasks (coffee beans before, dead bugs afterwards), participants in both conditions felt more aroused, *F*(2,118) = 51.18, *p*  <.001, η_*p*_^2^ = 0.47, and less pleased, *F*(2,118) = 90.00, *p* < .001, η_*p*_^2^ = 0.60, after the destruction of live bugs (see Fig. 3C). In addition, there was an interaction effect of *Condition* and *Destruction Task* with respect to arousal due to the high mean estimates of participants in the control group after the destruction of coffee beans, *F*(2,62) = 3.47, *p* = .037, η_*p*_^2^ = 0.10. Participants in both conditions felt less dominant after destruction of bugs (dead or alive) relative to the bean-destruction task, *F*(2,114) = 4.84, *p* = .010, η_*p*_^2^ = 0.08 (see Fig. 3C).

In sub-analyses for self-reported arousal and pleasure, the ANOVAs showed similar effects when only compliant participants were analyzed (i.e., 22 demand and 11 control condition participants): Those were more aroused, *F*(2,62) = 21.30, *p* < .001, η_*p*_^2^ = 0.41, and less pleased, *F*(2,62) = 56.61, *p* < .001, η_*p*_^2^ = 0.65, after the destruction of live bugs.

All other tests of the ANOVAs were above the significance level, *F*s ≤ 2.10 and *p*s ≥ .131.

### Questionnaires

Again, the means of the questionnaires were submitted to t-tests with condition as between-participants factor. Like in Experiment 1, results were above the statistical significance level and are accessible in the OSF repository.

### Tonic skin conductance levels (SCL)

The averaged tonic SCLs (time points) were submitted to two-factorial mixed-model ANOVA with *Condition* (demand, control) as between-participants factor and *Trigger event* (bug-destruction instruction, end of task, dead bug-destruction instruction) as within-participant factor. The ANOVA yielded a significant main effect of *Trigger event*, *F*(2,116) = 7.63, *p* < .001, η_*p*_^2^ = 0.116. Tonic skin conductance levels increased from the bug-killing instruction to the beginning of the dead-bug destruction task. The analysis also showed a significant interaction effect of the factors *Trigger event* and *Condition*, *F*(2,116) = 4.38, *p* = .015, η_*p*_^2^ = 0.070. Participants in the demand group showed a steady increase of tonic value over time while participants in the control group showed a relatively steady tonic value over time. There was no significant main effect of *Condition*, *F*(1,58) = 0.092, *p* = .762, η_*p*_^2^ = 0.002. As defiance was a main aspect of the study, we also conducted an exploratory ANOVA with the factors *Trigger event* and *Bug Destruction (yes/no)*, which resulted in a significant interaction, *F*(2,116) = 7.13, *p* < .001, η_*p*_^2^ = 0.109, with participants that chose to destroy the live bugs displaying a steady increase in tonic SCL (see also Fig. 3D).

If only SCL values of compliant participants are considered in the analysis (*n* = 32, demand condition = 21; control condition = 11), which constitutes a relatively small sample, the ANOVA only yielded a significant main effect of *Time*, *F*(2,60) = 11.79, *p* < .001, η_*p*_^2^ = 0.282, indexing an increase in tonic value over time irrespective of condition. All other *F*s ≤ 1.68 and *p*s ≥ .196.

### Agency and responsibility

The means of self-reported agency estimations and feelings of responsibility were submitted to two-factorial mixed-model ANOVAs with *Condition* (demand, control) as between-participants factor and *Destruction Task* (coffee beans, live bugs, dead bugs) as within-participant factor. Separate ANOVAs were computed with all participants and with the selection of compliant participants.

The ANOVA of participants’ agency estimations yielded a main effect of *Destruction Task, F*(2,118) = 11.23, *p* < .001, η_*p*_^2^ = 0.16. Participants estimated their active contribution to the destruction of live bugs smaller than that to the destruction of coffee beans and dead bugs, in line with the higher defiance rates for live bugs. The main effect of *Condition* was also significant, *F*(1,59) = 4.53, *p* = .038, η_*p*_^2^ = 0.07. Participants in the control condition estimated their active contribution lower than those in the demand condition. In the subanalysis of compliant participants (34 out of 61 participants), however, participants felt their active contribution to be higher for the destruction of live bugs relative to coffee beans and dead bugs, *F*(2,62) = 3.82, *p* = .027, η_*p*_^2^ = 0.11 (main effect of *Destruction Task*), and the main effect of *Condition* was not significant, *F* < 1 (see Fig. 3, Panel B).

The ANOVA with respect to participants’ feelings of responsibility yielded a significant main effect of *Destruction Task*: Participants felt less responsible for the destruction of live bugs than for that of coffee beans and dead bugs, *F*(2,118) = 10.39, *p* < .001, η_*p*_^2^ = 0.15. In the subanalysis of compliant participants (34 out of 61 participants), however, participants felt most responsible for the destruction of live bugs, *F*(2,62) = 4.82, *p* = .011, η*p*^2^ = 0.13 (see Fig. 3B).

All other tests were above the significance level, *F*s ≤ 1.92 and *p*s ≥ .171.

### Debriefing

No participant expressed suspicion that the destruction machine might be manipulated during the structured verbal debriefing. Some compliant participants even explicitly wished to see that the bugs were still alive.

## Discussion

The results of Experiment 2 replicated the results of Experiment 1 (except for the difference in self-reported feelings of dominance) and give additional insights into obedient conflict resolution. Compliant participants reported negative affect specific to the alleged destruction of live bugs, which qualifies their compliance as cases of obedience. Their physiological arousal, as indexed by tonic SCL, increased after alleged bug-killing irrespective of condition, whereas defiant participants’ physiological arousal remained steady. Nonetheless, compliant participants attributed high levels of agency and responsibility to themselves. Whereas the SCL data are compatible with both the notion of engaged followers^22^ and the notion of an agentic shift^4^, compliant participants’ high levels of self-reported agency and responsibility are incompatible with a ceding of responsibility to the experimenter as proposed by the latter account.

## General discussion

The present research tested the suitability of object-destruction paradigm (based on the bug-killing paradigm^29^) for the study of inner conflict that is characteristic of obedience (as a special case of compliance). The paradigm comprises several control conditions that allowed for investigating the motivational (obedience vs. defiance) and affective (pleasure, arousal, skin conductance) concomitants of the inner conflict elicited by an experimenter’s bug-killing demands as well as its resolution (skin conductance, agency, responsibility). Results of two studies revealed that participants were more willing to kill live bugs in an electric coffee grinder when demanded to do so (demand condition) in comparison to a condition in which they were reminded of their free will (control condition). Participants in the control condition also received more prods to proceed with the destruction task from the experimenter. Participants in both conditions reported more arousal and less pleasure during the bug-destruction relative to the respective other two destruction tasks. Self-reported feelings of dominance were lower during the destruction of bugs (dead or alive) relative to the destruction of objects (coffee bean, paper crane)—with (dis)obedience as a modulating factor in Experiment 1. In Experiment 2, participants tonic skin conductance levels (SCL) as well as responsibility and agency self-reports with respect to bug-killing were measured in addition, allowing for insights into obedient participants’ conflict resolution. After bug destruction, SCLs of compliant participants increased, whereas defiant participants’ SCL levels did not increase. Moreover, compliant participants reported higher estimates of active contribution and responsibility for the destruction of live bugs relative to the destruction of inanimate objects (coffee beans, dead bugs).

The present research aimed at solving a double dilemma: (1) Confronting participants with an immoral demand that is ethically acceptable; (2) showing that many among them experience an inner conflict in response but still comply, thus qualifying their compliance as obedience^4^. The results suggest the object-destruction paradigm to be a promising candidate for overcoming this challenge: Both the differential compliance and verbal resistance of participants from the demand vs. control group imply that most participants did not want to kill the bugs and/or did so with personal reservation. In the control group, most participants needed an explicit prod from the experimenter to proceed with the destruction of bugs. Moreover, affective judgments of the three destruction tasks indicate that the instruction to kill live bugs and/or the alleged killing of bugs was negatively arousing in comparison to the other tasks, irrespective of the condition. Overall, these motivational and affective effects indicate that the experimenter's instruction to kill bugs produced an inner conflict in most participants^4,22^. Thus, the results of the present research suggest that the object-destruction paradigm is suitable for the study of the conflict experience and resolution underlying obedience (vs. defiance) to authority. Furthermore, the results also suggests that the bug-killing task is suitable for the study of obedience and its mechanisms in general, as conflict experience is a crucial prerequisite for obedience^4^ as opposed to other types of compliance (e.g., compliance with a small request from a neighbor^6^).

Experiment 2 additionally elucidated obedient (vs. defiant) participants’ conflict resolution in response to the bug-killing demands in more detail. The increasing physiological arousal of compliant but not defiant participants indicates that the former group’s initial self-reported negative arousal intensified over time. Moreover, compliant participants gave higher responsibility and agency estimates following bug- relative to object-destruction, which indicate that participants felt more causally involved in killing bugs relative to the other tasks^cf.^^23^. Note that values were very close to the maximum of the respective scales with many participants choosing the maximum value^49^. This finding is incompatible with an explanation based on a transfer of responsibility to the experimenter as suggested by the agentic-shift account^4^. In sum, these results indicate that many compliant participants felt badly about joining in with the experimenter’s bug-killing demands; but still did so to the effect of perceiving agency and responsibility.

The question of which factors and/or processes are causally responsible for obedience vs. defiance in the face of destructive authority continues to be a topic of debate, even in recent articles on the subject^18,24,26,53,54^. Adding to this ongoing discourse, our research demonstrates that emphasizing the significance of one’s free decision-making within the context of the experimenter’s instructions to destroy bugs led to a decrease in obedience. As a result, individuating the decision nudged conflict resolution toward acting in line with one’s own values and norms rather than at the behest of the experimenter. The more pressing question, however, is what motivational force drove obedient participants to submit to the experimenter’s demands, even to the point of ignoring their inner disapproval of taking the bugs’ lives. The results of the present research are difficult to reconcile with a shift from a self-directed mode to an ‘agentic state’ that relieves participants from responsibility^4^, but could be compatible with a process of social persuasion to the effect of engaged followership^55^. Looking at the video recordings of Experiment 1, though, it appears that during the bug-destruction task, most obedient participants felt somehow obligated to do as the experimenter said^4,21^ rather than to internalize his purported goal ^e.g.,^^56^ to the effect of happy companionship^22^. Moreover, in both conditions, the prod that appealed to the scientific purpose of the experiment persuaded only few participants to kill the bugs.

As an alternative to the persuasion process implied in the engaged followership account^22^, we propose to consider a recent reconceptualization of the sense of obligation^57^ that is rooted in joint action theorizing^58–60^ as missing link between social identification and obedience. Here, a sense of obligation is associated with coercive qualities as it emerges in the context of consensual and interdependent joint action among two or more people. This type of interaction is characterized by *shared* expectations that each co-actor feels compelled to fulfill. Moreover, in the context of obedience, the joint experimenter-participant action is further qualified by a leader–follower role asymmetry^61,62^ between the two that favors the experimenter. In line with this reasoning, the obedient participants’ commitment to play their destructive part^49^ might have been driven by a sense of *joint* agency^63^ that made it difficult to disappoint the expectations they *shared* with the experimenter relative to their *individual* moral reservations. Notably, this theorizing is compatible with a critical role of social identification with the experimenter and does not exclude the possibility that obedient individuals evaluate their contribution more positively at a later point in time^55^. However, since the present research did not test the persuasion process^22^ or possible joint action underpinnings, an investigation of the exact nature of the underlying process(es) of obedience is a task for future research.

The present research also has important limitations. Firstly, participants in the control condition had to reject the experimenter’s instructions and two prods before the destruction task was aborted. Consequently, even some of the participants that decided to comply with the bug-killing instructions might have felt pressured to do so. Nonetheless, some participants in the control groups killed bugs without a prod. If these ‘willing followers’ also felt pressured, were persuaded by the experimenter, just did not bother to kill bugs, or even enjoyed the bug-destruction task^64^ is to be determined in future research. Secondly, participants’ estimates of responsibility/agency were measured with unipolar scales focusing on the individual alone whereas previous research had used a multipolar instrument^49^. More precisely, participants there had been allowed to allocate responsibility in percent between themselves, the experimenter, and the victim. Thirdly, most participants in both experiments identified as female. In future experiments, efforts could be made to recruit a more gender-balanced sample of participants. Finally, the number of compliant participants in the obedience condition differed between Experiments 1 and 2, which we explain with the change of experimenters, the drop of video recording^65^, and/or other factors to be investigated in future research.

Experiencing inner conflict in response to an authority figure’s immoral demands but yielding to it is what differentiates obedience from compliance^4,6^. To shed light on this conflict and its resolution, the present research tested an ethically acceptable alternative to the Milgram paradigm^7^ for the study of obedience to authority. The ‘object-destruction paradigm’ adds two types of control conditions to the bug-killing task^29^: A free-decision control group that is asked rather than demanded to kill bugs in an electrical coffee grinder; and control destruction tasks (e.g., coffee beans) that allow for direct comparison regarding affective response characteristics. Two experiments showed that more participants were willing to kill the bugs in the demand than the control group. All participants self-reported more negative arousal when instructed to allegedly destroy live bugs relative to other objects. Experiment 2 allowed for insights into obedient participants’ conflict resolution. Tonic skin conductance measurements indicated that obedient participants’ negative affective response lasted longer than that of deviant participants. Crucially, they self-reported high levels of agency and responsibility for bug-killing, nonetheless. Overall, the present research findings are at odds with explanations of obedience based on a transfer of responsibility^4^, whereas they are in line with explanations based on a sense of obligation^57^ and potentially engaged followership^22^. Finally, the present research offers important ethical implications for psychological science in general: Firstly, participants might feel obliged to an initial agreement even to the extent of working against their own interests (if not well-being). Secondly, researchers should be careful not to confuse the absence of resistance with an expression of one’s own ‘free will’.

## Data Availability

Documents, experiment program files, and all datasets generated and analyzed in Experiments 1 and 2 as well as the exact instructions (and/or their English translations) of the experimenters, the research protocols, detailed transcripts of the video-recorded bug-destruction tasks, and anonymized example videos of both conditions are available in an Open Science Framework (OSF) repository: https://osf.io/ekrj4/. For data protection reasons, the video recordings of Experiment 1 and the skin conductance level (SCL) recordings of Experiment 2 are available from the corresponding author on reasonable request. In accordance with the participants’ informed consent declarations regarding data processing, editors and reviewers had access to the video recordings of the bug-destruction task of Experiment 1 during peer review.
